# Computational Investigation of the Structural Properties of Poly(lactic acid) and Its Stereoisomers: Shape, Size, and Flexibility

**DOI:** 10.1021/acs.macromol.5c00918

**Published:** 2025-07-15

**Authors:** Petra Bačova, Vagelis Harmandaris, Sergio I. Molina

**Affiliations:** † Departamento de Ciencia de los Materiales e Ingeniería Metalúrgica y Química Inorgánica, Facultad de Ciencias, IMEYMAT, Universidad de Cadiz, Campus Universitario Río San Pedro s/n., Puerto Real, Cádiz 11510, Spain; ‡ Computation-Based Science and Technology Research Center, The Cyprus Institute, 20 Constantinou Kavafi Str., Nicosia 2121, Cyprus; § Institute of Applied and Computational Mathematics (IACM), Foundation for Research and Technology Hellas (FORTH), Heraklion, Crete 70013, Greece; ∥ Department of Mathematics and Applied Mathematics, 37777 University of Crete, Heraklion, Crete 71409, Greece

## Abstract

In an effort to support the common cause of making the polymer industry more sustainable, studies involving computational methods have been enriched with polymers of natural origin. Owing to their complex structure often resembling proteins more than synthetic polymers, simulation studies of biobased polymers are very challenging. In this work, we present an atomistic study of poly­(lactic acid) and its stereoisomers of multiple molecular weights in the melt. We show that while the L- and D-stereoisomers exhibit similar global structural properties, copolymer chains consist of more compact and more spherical domains than their homopolymer analogues. We investigate the local folding in these structures by examining intramolecular hydrogen bonding, packing length, and the geometrical parameters characterizing the n−π* interaction. This work provides a detailed description of the inner structure of this biodegradable polymer over various molecular weights and reveals its structural similarities with synthetic and biopolymer materials.

## Graphical abstract

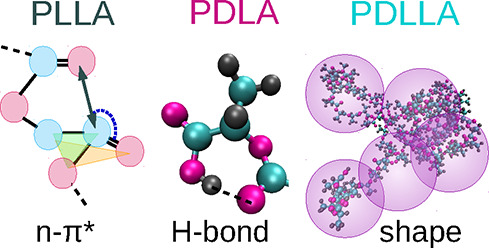

## Introduction

With sustainability being one of the main goals of the policies on research and innovation strategies,^1^ the interest of the polymer community turned to biobased polymers and more sustainable practices. One family of polymer materials complying with the new regulations is the group of biodegradable polymers with application in additive manufacturing. Additive manufacturing is considered more environmentally friendly than conventional manufacturing methods due to its reduced waste and carbon dioxide production.^2^

Poly­(lactic acid) (PLA) is among the most popular polymers with application in additive manufacturing, as due to its low extrusion temperature, it is a user-friendly material for printing customized objects at a small scale.^3^ PLA shares some similarities with common synthetic polymers;^4^ however, its industrial usage has some drawbacks, which have been addressed by a wide range of experimental studies.^5,6^ PLA is biodegradable under certain conditions,^4^ and due to the presence of a chiral center, there are two types of monomers, L and D, and therefore two types of homopolymers, PLLA and PDLA, as shown in Figure 1a. These optical isomers of lactic acid-based polymers can also be labeled with (*R*)- and (*S*)-stereodescriptors; here, we keep the L and D notation, as it is widely used in commercial jargon. The structural properties of PLLA have been mostly studied in crystalline form and in solutions,^7,8^ and while studies of PDLA are much less common, there are some recent indications of substantially different conformational behavior of PDLA in comparison to a random copolymer with a 1:1 molar ratio of d-lactide to l-lactide in solution.^9^ Theoretical studies of conformational properties encompass PLLA and its copolymers;^10,11^ however, they are often based on structural characteristics extracted from oligomers and/or small organic molecules. Interestingly, PLA stereocomplexes, created by the cocrystallization of the enantiomeric PLLA and PDLA, have attracted a lot of attention recently, mainly due to their peculiar properties, such as a higher melting point in comparison to the homocrystals.^12^

**Figure 1:**
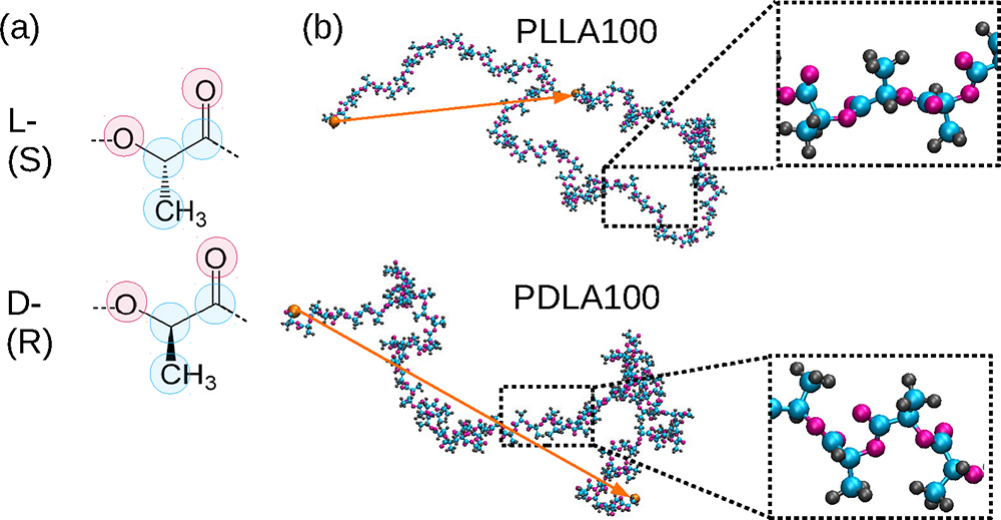
(a) Sketch of a monomer with L and D stereochemistry. (b) Randomly selected snapshots of poly­(lactic acid) consisting of 100 monomers. The orange solid arrow indicates the end-to-end distance. The terminal hydrogens are represented by large orange spheres for better visibility.

Concerning the computational approaches studying PLA and its properties in melt, more specifically, approaches employing atomistic and coarse-grained simulations, they struggle to keep pace with experimental studies, mainly due to the structural and dynamical complexity of PLA. This complexity is reflected in the long relaxation times of the structural properties such as the radius of gyration *R*_g_ and makes the equilibration procedure in the limit of current computational feasibility.^13,14^ For an extensive summary of the field, we refer readers to a recent review.^15^ Here, we would like to stress that the majority of the computational studies focus on PLLA and that, to the best of our knowledge, there are no simulation studies of pure PDLA. Concerning the effects of stereochemistry, the recent work of Guseva et al.^16^ highlights the decrease in the radius of gyration and in the values of the characteristic ratio with increasing content of the D-stereoisomer in copolymer systems.

Detailed analysis of molecular shape, size, and, in general, the local packing plays an important role in the characterization of biological macromolecules, such as proteins and/or DNA.^17–19^ These characteristics help identify functional specificity as well as the effect of the intramolecular interactions on the optimal spatial arrangements of these molecules, e.g., during complexation with other objects. To that end, they have also been investigated in common synthetic polymers of complex topology, such as single-chain nanoparticles,^20^ star-shaped molecules,^21^ or rings.^22^ It should be noted that, in the case of polymer systems, information about local packing is widely used in the design of mesoscopic models.^22–25^

Here, we present a detailed atomistic investigation of the structural properties of PLA and its stereoisomers (PLLA, PDLA, and their copolymers) of multiple molecular weights. Our aim is two-fold: (i) to provide a detailed description of the inner structure of this biodegradable material in order to reveal its similarities with synthetic and biopolymer materials and (ii) to facilitate the bridging of the theoretical knowledge published so far with experimentally relevant length scales by investigating chain-length effects. In (i), we seek to systematically address the differences in folding of different stereoisomers and thus contribute to the understanding of the structure–properties–performance relationship in these materials, without facing the experimental challenges common for PLA materials such as partial crystallinity, degradation, and/or polydispersity. More specifically, by investigating both homopolymers, PLLA, and PDLA, we aim to detect the structural features coming from both homopolymers in the copolymer materials, which have been mostly compared computationally to the PLLA case. Concerning point (ii), we focus on detecting the molecular weight limit at which the chain exhibits Gaussian behavior, as chain-length effects are one of the common causes of discrepancies when applying theoretical models to interpret experimental as well as simulation data. To achieve these objectives, we study the shape and other single-molecule structural properties in these macromolecular systems and focus on the effect of specific intramolecular interactions on the final molecular conformation in the melt.

## Methods and Simulation Details

We simulated two different chains lengths of the D-stereoisomer of PLA (PDLA), six different molecular weights of the L-stereoisomer of PLA (PLLA), and its two random copolymers with the content of the D-monomer of 16 and 55%. Multiple molecular weights of the homopolymers were selected to investigate the chain-length effects in systems ranging from oligomers to approximately entangled molecular weight. The copolymer system with 55% of the D content is the result of a random process of selecting the D- or L-monomer along the chain, during which we sought a balanced amount of both types of monomers as it would be the case during the production of PLA by the polycondensation of racemic lactic acid (equal amounts of L-lactic acid and D-lactic acid). Experimentally, PLA with a low content of the D-monomer (<2%) is considered highly crystalline, while copolymers with a D content higher than 12% are generally amorphous;^26^ the copolymer with 16% of the D content was selected to fall close to this generic experimental limit. Graphical illustrations of the given stereochemistry and of a randomly selected configuration of the PLLA and PDLA chains are shown in Figure 1a,b, respectively. Each simulated system consists of 70 polymer chains, and the corresponding box size is bigger than the average end-to-end distance *R*_e_. The studied molecular weights together with the labeling of the systems and some of their characteristics are listed in Table 1.

**Table 1:** Labeling of the Simulated Systems Together with Their Compositions and Some Characteristics^a^

system label	*N*	L- [%]	D- [%]	*M* [g/mol]	*a*	*R*_e_ [nm]	*p* [nm]	ρ [kg/m^3^]
PLLA10	10	100	0	738.7	0.508 ± 0.002	2.040 ± 0.005	0.266 ± 0.002	1110 ± 10
PLLA30	30	100	0	2179.9	0.49 ± 0.01	4.11 ± 0.08	0.192 ± 0.004	1117 ± 5
PDLA30	30	0	100	2179.9	0.46 ± 0.01	3.77 ± 0.09	0.229 ± 0.006	1110 ± 5
PLLA100	100	100	0	7224.4	0.479 ± 0.003	8.4 ± 0.1	0.152 ± 0.002	1121 ± 5
PDLA100	100	0	100	7224.4	0.421 ± 0.008	7.35 ± 0.05	0.200 ± 0.002	1113 ± 4
PLLA125	125	100	0	9026.0	0.423 ± 0.005	8.7 ± 0.1	0.176 ± 0.002	1122 ± 5
PLLA150	150	100	0	10827.6	0.414 ± 0.005	9.8 ± 0.1	0.167 ± 0.002	1122 ± 4
PLLA175	175	100	0	12629.2	0.438 ± 0.006	10.6 ± 0.2	0.166 ± 0.003	1122 ± 4
copo16D	100	84	16	7224.4	0.42 ± 0.01	6.6 ± 0.1	0.246 ± 0.004	1119 ± 6
copo55D	100	45	55	7224.4	0.376 ± 0.008	5.41 ± 0.08	0.368 ± 0.006	1115 ± 6

a *N* stands for the number of monomers in the chain, *M* for the molecular weight per chain, *a* for the average asphericity, *R*_e_ for the average end-to-end distance $R_{e}=\langle R_{e}\rangle =\sqrt{\left\langle R_{e}^{2}\right\rangle }$ , *p* for the packing length, and ρ for the average density of the system. The error bars for *a* and *R*_e_ were obtained by dividing the simulation trajectory into four blocks and using a standard block averaging method.

All simulations were performed by an open-source software GROMACS.^27^ All-atom representation with explicit hydrogens and the PLAFF3 force field^28^ was chosen to model the chains under study in an amorphous state. The tabulated potential used in the force field published in ref 28 to describe one of the backbone dihedrals was replaced with a functional form corresponding to the Ryckaert–Bellemans function^29^ in order to be able to model copolymer chains (see Figure S1 in the Supporting Information). To have a consistent set of data for a fair comparison, this potential was also replaced in the homopolymers. The details about this modification are given in the Supporting Information. The bonds were constrained by the LINCS algorithm.^30^ The preparation and the equilibration of the system are described elsewhere.^14^ Here, we only briefly mention that the GROMACS configurations published in ref 28 were taken as the primary source of a PLLA chain configuration, and a homemade code for changing the stereochemistry was applied to obtain the initial PDLA and copolymer configurations. The code for changing the stereochemistry and the procedure used to shorten the chains published in ref 28 are publicly available.^31^ The equilibration consisted of a series of short *NPT* runs to adjust the density, a consecutive run at 600 K to promote intermixing of the chains, a cooling run, and a final equilibration run at 500 K, which lasted from 100 ns in the case of the shortest PLLA10 up to 2 μs in the case of the longest PLLA175 chains. After the equilibration, each system was simulated for an additional 1 μs at conditions identical to the production run. Namely, the temperature of 500 K was maintained by the Nose–Hoover thermostat, the pressure of 1 atm was regulated by the Parrinello–Rahman barostat, and the particle mesh Ewald method was used for the electrostatic interactions. The time step was 2 fs for the PLLA and copolymers and 1 fs for the PDLA systems. The production run, during which the data for analysis were collected, lasted 1 μs.

## Results and Discussion

In order to characterize the conformational behavior of the studied polymers, we calculated some of the fundamental static properties. The radius of gyration *R*_g_ of the molecule consisting of *N*_a_ atoms is defined as

$$\langle R_{g}^{2}\rangle =\frac{\sum_{i}^{N_{a}}m_{i}(r_{i}-r_{\mathrm{CM}}{)}^{2}}{\sum_{i}^{N_{a}}m_{i}}$$ 1

where *m*_*i*_ and *r*_*i*_ represent the mass and the position of the atom *i*, respectively, and *r*_CM_ represents the position the center of mass of the given molecule. In our systems, the number of atoms can be calculated as *N*_a_ = 9­(*N* – 2) + 10 + 11, since the middle monomer consists of nine atoms (see Figure 1a), and the terminal monomers contain a carboxyl and a hydroxyl group typical for esters. When referring to *R*_g_, we refer to $R_{g}=\langle R_{g}\rangle =\sqrt{\left\langle R_{g}^{2}\right\rangle }$ . The data obtained from the simulations are compared to the literature data in Figure 2a and are listed in Table S1 in the Supporting Information. It should be noted that, apart from the different temperatures labeled in the legend of the Figure 2, the data published in refs 16 and 23 were obtained for model systems employing different force fields; therefore, a quantitative agreement is not expected. Nevertheless, it is worth pointing out certain generic features. The collected data seem to follow the scaling prediction of *R*_g_ ∼ *N* ^0.5^, expected for polymers in melt, except for the shortest chains, namely, 10mer PLLA in our study and in the study by Prasitnok^23^ and the 13-mer published in previous studies.^16,24^ A weak deviation for different temperatures is observed. It should be noted that PLA is a semicrystalline polymer; therefore, there might be some crystalline regions present at 298 K in ref 23. In addition, both studied copolymers seem to exhibit smaller values of *R*_g_ in comparison with their homopolymer analogues. The same trend was reported for random copolymers in ref 16.

**Figure 2:**
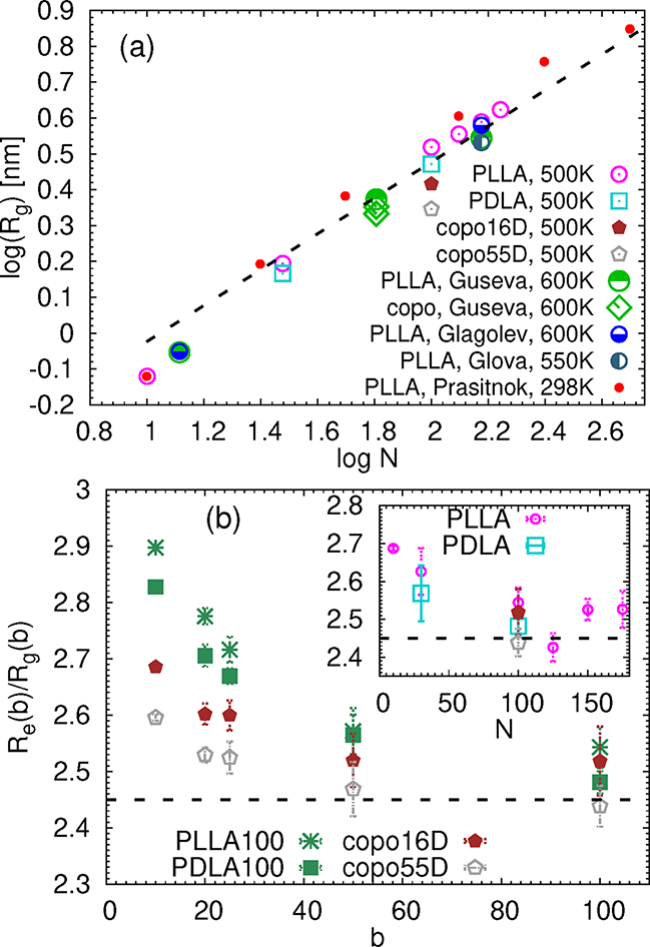
(a) Radius of gyration *R*_g_ as a function of the number of monomers per chain *N*. The dashed line indicates $R_{g}∼\sqrt{N}$ scaling. The data published at different temperatures, i.e., at 600 K in ref 16 and in ref 24, at 550 K in ref 13, and at 298 K in ref 23 are also added. Note that the data for the random copolymers from ref 16 overlap (i.e., there are four yellow circles on the top of each other). The error bars are on the order of the symbol size. (b) Ratio *R*_e_/*R*_g_ as a function of the number of monomers *b* in the subdomain for chains containing 100 monomers. Inset shows the same quantity for *b* equal to the full length of the chain, *N* and for all of the studied systems. The dashed line represents the theoretical value for an ideal chain. The standard block averaging method was used to obtain the error bars.

Concerning the local conformation of the chains and their Gaussian character, we calculated the ratio of the end-to-end distance to the radius of gyration of selected parts of the chain. Namely, in Figure 2b, we divided the chains into sections of *b* monomers and compared the ratio *R*_e_(*b*)/*R*_g_(*b*) to the theoretical value $\sqrt{6}$ , derived for the ideal chains in melt.^32^ Comparing the chains with different stereochemistry, the PLLA chains deviate the most from the theoretical behavior at length scales *b* < 100, while the copolymer with 55% of the D content behaves as an ideal chain for *b* ≥ 50. A similar trend is observed for the ratio of *R*_e_(*b*)/*R*_g_(*b*) where *b* is equal to the total number of monomers in the chain, *N* (see the inset of Figure 2b). Namely, the values for PLLA are systematically higher than those for PDLA and/or for the copolymers. In addition, for PLLA, the value indicating the ideal behavior seems to be reached for *N* = 125. This observation is in agreement with the results published in ref 33, where the value *R*_e_^2^/(6*R*_g_^2^) = 1.0 ± 0.1 was found for PLLA chains of 150 monomers. The ratio *R*_e_^2^/(6*R*_g_^2^) calculated for PLLA150 falls in this range of values. It should be noted that a division into sections mimics the theoretical concept based on a blob-like picture, in which the blobs are of the size of the Kuhn length.^32^ Having this concept in mind, the results in Figure 2b indicate possible differences in local flexibility among the studied stereoisomers.

In order to quantify the flexibility of the chains, we calculated the characteristic ratio *C*_*∞*_ as

$$C_{\infty }=\lim_{n\to \infty }\;R_{n}^{2}/(nl_{b}^{2})$$ 2

where *n* is the number of bonds along the backbone (*n* ≈ 3*N*), and *l*_b_ is the average bond length. As the backbone of each chain consists of different types of bonds (see Figure 1), we calculated *l*_b_ as the average length of all bonds included in the backbone, giving the value of *l*_b_ ≈ 0.138 nm. In eq 2, *R*_*n*_ represents the average internal distance between two backbone atoms *i* and *i* + *n*, namely, *R*_*n*_^2^ = ⟨(|*r*_*i*_ – *r*_*i*+*n*_|)^2^⟩, where ⟨⟩ denotes the average over all chains and time frames, and *r*_*i*_ and *r*_*i*+*n*_ are the positions of the backbone atoms separated by *n* bonds. The value of the characteristic ratio is *n-*dependent, which can be denoted as *C*_*n*_. In other words, short chains appear to have lower values of *C*_*∞*_; therefore, if the Kuhn or the persistent length is calculated for those chains, they appear to be more flexible than chains of a high molecular weight of the same polymer type. It should also be noted that these chain-length effects are present when theoretical models assuming Gaussian character are applied in the estimation of the persistence length from experimental and/or simulation results of systems, which are not long enough to fulfill this condition.

In Figure 3, the *n*-dependent characteristic ratio *C*_*n*_ is plotted for all of the studied systems. As seen also in the inset of Figure 3a, *C*_*n*_ is an increasing function of *n* for PLLA10 and PLLA30. For longer PLLA chains, particularly for *N* ≥ 125, the function reaches a plateau around *n* ≈ 100, which would correspond to *N* ≈ 33. The plateau observed in our systems is in agreement with the previously published results on PLLA (shaded region in Figure 3a) at different temperatures, namely, *C*_*∞*_ = 10.2 ± 0.2 for *N* = 150 at 550 K^13^ and *C*_*n*_ ranging from 10.31 ± 0.04 for *N* = 13 to 10.65 ± 0.06 for *N* = 64 at 600 K.^16^ Concerning PDLA and copolymer chains, the dependence of internal distances on the backbone atom separation in PDLA and copolymer chains closely follows the dependence of PLLA chains of the same length up to a certain point (corresponding to *n* ≈ 10 and thus *N* ≈ 3) at which differences in monomer packing prevail. It should also be noted that when comparing data in Figure 3a,b, the plateau defining *C*_*∞*_ is well-developed for PLLA125, while in the case of the PDLA systems, the data for shorter, PDLA100 chains seem to reach the limiting behavior. Interestingly, and in accordance with the observations commented above, while PDLA seems to be of a similar flexibility as PLLA (*C*_*∞*_ ≈ 9), the copolymers exhibit much lower values of *C*_*∞*_ in comparison to their homopolymer analogues, with the *C*_*∞*_ being smaller for higher content of the D-monomer. The same conclusions were reached when analyzing the data from the correlation functions of the vectors along the backbone and in terms of the worm-like chain model (see Section 2 in the Supporting Information). This general tendency agrees well with the data reported in the literature via experimental studies and simulations, even though the actual values of *C*_*∞*_ are a point of debate for decades. We refer the readers to the experimental work,^34^ which lists possible reasons of discrepancies among different studies, and/or to a recent review, which summarizes both types of work and provides deeper insight into the issue.^15^ It should also be noted that, to the best of our knowledge, there is not much known about the conformational behavior of pure PDLA, except for a few studies determining radius of gyration from the solution.^9,35^

**Figure 3:**
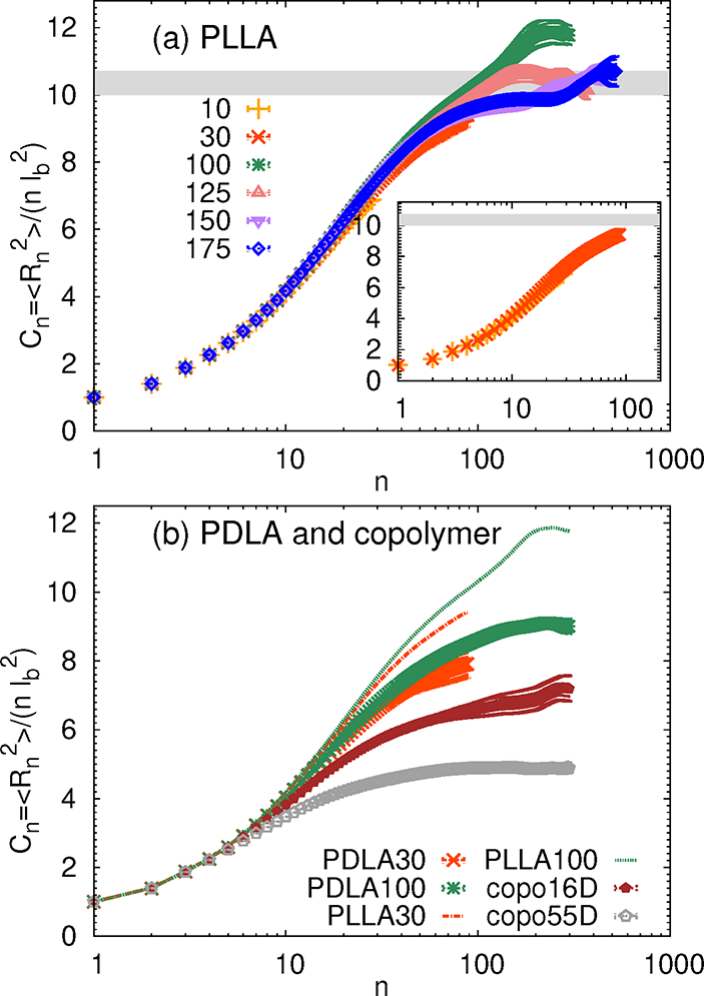
Characteristic ratio *C*_*n*_ for (a) PLLA and (b) PDLA and copolymer systems as a function of the distance along the backbone, *n*, where *n* ≈ 3*N*. The inset in (a) shows data for PLLA10 (crosses) and PLLA30 (squares). The shaded region in (a) indicates the range of *C*_*n*_ values obtained for chain lengths from *N* = 13 to *N* = 150 for 600 K in ref 16 and for *N* = 150 at 550 K in ref 13. The standard block averaging method was used to obtain the error bars.

The flexibility of the chain is closely connected to the packing length, whose values are reported in Table 1. The quantity was calculated from the data in the table as

$$p=\frac{M}{\langle R_{e}^{2}\rangle \rho N_{A}}$$ 3

where *N*_A_ denotes Avogadro’s constant. Given the fact that the density dependence on *N* is minimal in our systems (see Table 1), the packing length inversely reflects the dependence of the polymer size, expressed in the form of *R*_e_, on the molecular weight of the chain and thus on *N*. Comparing the values with the values reported for common synthetic polymers (see also Figure S4 in the Supporting Information), the data for our systems lie in the range of values typical for flexible polymers with the backbone containing mainly carbon atoms, such as polyethylene and polybutadiene^36^ or one heteroatom such as poly­(ethylene oxide).^37^ It should be noted that this comparison is orientative because the values of *p* slightly fluctuate depending on the source of experimental data (compare, e.g., refs 36 and 37). Nevertheless, as the packing length is the parameter relating equilibrium chain dimensions and entanglement behavior, it is interesting to observe that our PLA systems are closer to the behavior of polymers that lack specific stereochemistry, rather than polymers such as poly­(methylmethacrylate), which is optically active.^37^

Following with the spatial arrangement of the molecules, we investigate the shape of the studied polymers by calculating the geometrical inertia tensor:

$$T_{\alpha \beta }=\frac{1}{2N_{a}^{2}}\sum_{i}^{N}\sum_{j}^{N}(r_{i\alpha }-r_{j\alpha })(r_{i\beta }-r_{j\beta })$$ 4

where α, β denote the *x*, *y*, *z* components of the Cartesian coordinates. The eigenvalues of *T*_αβ_, labeled as λ_1_, λ_2_, and λ_3_, were used to calculate the asphericity parameter, defined as

$$a=\frac{(\lambda_{2}-\lambda_{1}{)}^{2}+(\lambda_{3}-\lambda_{1}{)}^{2}+(\lambda_{3}-\lambda_{2}{)}^{2}}{2(\lambda_{1}+\lambda_{2}+\lambda_{3}{)}^{2}}$$ 5

The values of the asphericity parameter range from 0 to 1, with the most spherical objects having values of *a* equal to 0. The distributions of the asphericity parameter for all PLLA systems are shown in Figure 4a, and the average values are listed in Table 1. As the chain length increases, the maximum point of the distribution shifts closer toward 0, indicating that the longer the chain, the more spherical is the molecule. The same tendency is observed for PDLA chains in Figure 4b. In addition, PLLA and PDLA chains of the same length seem to be similar in shape. Concerning the copolymer chains, the distribution of the asphericity of the system with 16% of the D content closely follows the data for PLLA and PDLA chains, while the system containing 55% of the D-monomer appears to be slightly more spherical.

**Figure 4:**
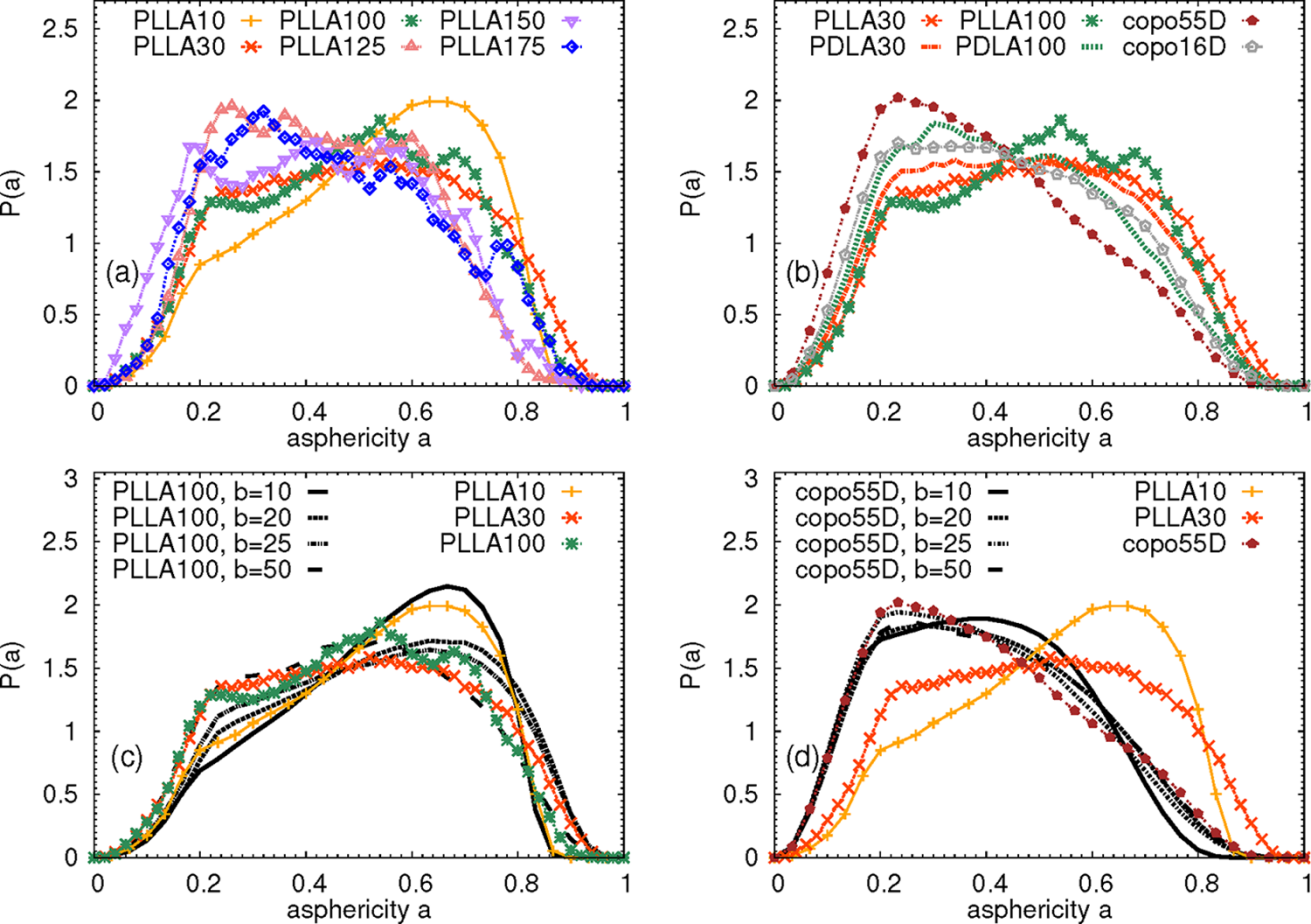
Asphericity of the simulated systems. (a) Probability distribution functions of the chain asphericities for PLLA systems. (b) Probability distribution functions of the chain asphericities for PDLA and copolymer systems in comparison to the PLLA chains of the same length. (c) Asphericities of the PLLA chains with 10, 30, and 100 monomers in comparison with the asphericities of the domains in PLLA100 containing *b* monomers. (d) Asphericities of the PLLA10, PLLA30, and copolymers with 55% of the D content in comparison with the asphericities of the copolymer domains containing *b* monomers.

In Figure S5 of the Supporting Information, we present the comparison of our data with the data collected from the literature. Common unentangled synthetic polymers in melt such as polystyrene (PS), polybutadiene (PB), and poly­(ethylene oxide) (PEO) seem to exhibit a very similar shape as those observed in our simulations (see Figure S5b in the Supporting Information). In addition, our data for the longest studied chain, PLLA175, resemble the data obtained for a mesoscopic model of a chain capable of forming reversible bonds. The percentage of the reactive monomers in that system is low, as it is the case in the PLA polymers, and apparently the distribution of *a* does not differ significantly from the one for the chains without the capacity of hydrogen bonding (compare Figure S5a,b).

Considering the local behavior of the chains, we also investigate the spherical character of the domains along the chain. The data shown in Figure 4c,d confirm the trend observed in Figure 4a,b. As the size of the domain increases in PLLA systems, the domain becomes more spherical. The same tendency was observed for PDLA polymers (data not shown). Interestingly, the domains in copolymer system copo55D are rather spherical, independently of their size, which indicates a rather different mutual orientation of the adjacent monomers, which form the domains, than in the homopolymer chains. Very similar behavior was found for copo16D (data not shown), with the domains slightly less spherical than in copo55 but systematically more spherical than in homopolymers.

We investigated this mutual orientation by calculating the geometrical parameters defining the n−π* interaction (see Figure 5a). No clear evidence of the presence of n−π* interactions was found in our systems; however, the parameters shown in Figures 5b and S6 of the Supporting Information reveal different mutual orientations of the adjacent monomers in the two types of homopolymers, despite their very similar flexibility and shape. The most probable mutual position of two PLLA monomers is such that the distance between the oxygen and the carbon in the adjacent carbonyl groups is equal to the Lennard-Jones diameter of their nonbonded interaction (see the double dashed gray vertical line in Figure 5b), while in the case of PDLA, the two monomers are further apart. As shown in Figure S6c, the distributions of the distances for the L–L and D–D pairs of monomers in copolymers show some discrepancies with respect to the distributions of the reference homopolymers, which means that the mutual positions of the adjacent monomers are not only affected by the stereochemistry of the given monomers but also by the heterogeneous local environment (i.e., by the presence and sequence of neighboring monomers of both types).

**Figure 5:**
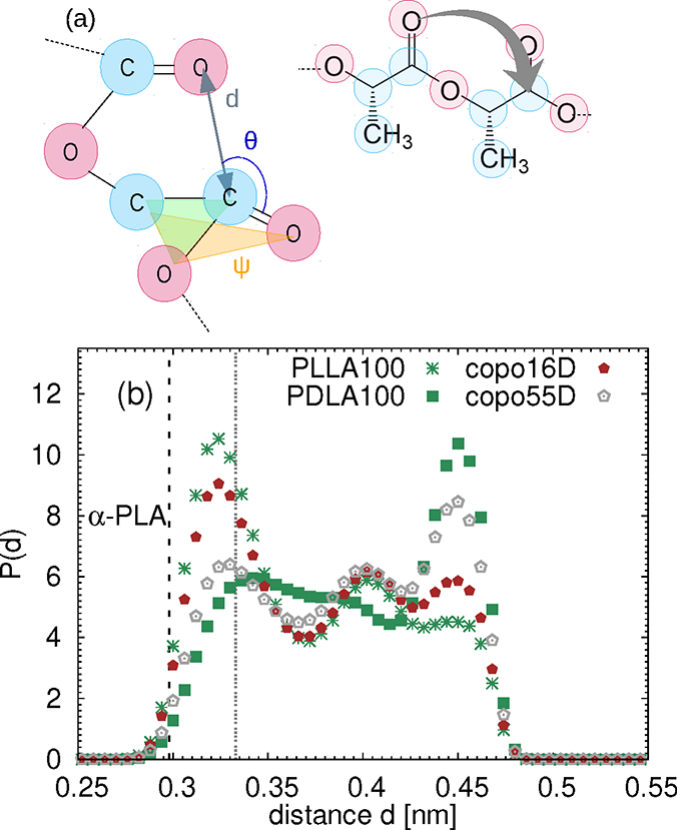
Geometrical parameters defining the n−π* interaction. (a) Sketch illustrating the interaction among the carbons and oxygens in the neighboring ester groups. The angle ψ is the angle between two planes highlighted by orange and green color. (b) Probability distribution functions of the distance d for the studied systems. The dashed vertical line indicates the value reported for the α conformational isomer of PLA in ref 8. The double dashed gray vertical line corresponds to the Lennard-Jones diameter of the carbon–oxygen interaction between the two consecutive carbonyls.

Another way to examine the local packing inside of the molecule is to measure the single-molecule form factor, defined as

$$W(q)=\langle \frac{1}{N_{a}}\sum_{j,k}^{N_{a}}\frac{\sin(qr_{jk})}{qr_{jk}}\rangle$$ 6

where *q* is a wave vector, *r*_*jk*_ is the distance between atoms *j* and *k*, and the brackets denote an average over all molecules and time frames. Due to the fractal nature of the polymers, the form factor typically exhibits a decaying power law dependence, *W*(*q*) ∼ *q* ^–1/ν^, in a window of wave vectors corresponding to length scales bigger than the bond length *l*_b_ but smaller than the radius of gyration, *R*_g_, i.e., 1/*l*_b_ ≳ *q* ≳ 1/*R*_g_. The form factors for PLLA and PDLA chains of the same molecular weight perfectly overlap, as can be seen in Figure 6, strengthening the aforementioned statements about their similar overall structural behavior. The exponent ν = 0.58 found for these systems is closer to the Flory exponent for a self-avoiding walk, (ν_SAW_ = 0.58) than to the one expected for ideal chains in the melt, i.e., (ν_IC_ = 0.5), which indicates that the chains are slightly more extended than those in which the excluded volume is fully screened. The same exponent was found for linear chains capable of forming reversible bonds at densities above the percolation threshold, which contained only a low percentage of the reactive sides and therefore might be seen as a simple model for polymers with a weak probability of forming hydrogen bonds.^38^ This behavior was attributed to the higher probability of formation of short-range than long-range intramolecular bonding (i.e., short “loops” between two connected species), together with the favorable formation of intermolecular over intramolecular bonds. In the case of copolymer chains, the exponent is slightly lower (see Figure 6b), ν = 0.48, which is in line with the more globular (partially reflected also in the average values of asphericity in Table 1) and more flexible character of these materials commented before. It should also be noted that while the deviation in slope in the comparison of the homopolymer and copolymer systems is obvious, the actual values of the exponent, namely, their second decimal place, are sensitive to the range of *q* vectors in which they are estimated. Having this limitation in mind, our data are also in acceptable agreement with those found for unentangled atactic polystyrene and poly­(ethylene oxide) chains (data not shown here, but are identical to the *W*_intra_ from ref 39), with an exponent ν = 0.5. It is also worth mentioning that these two synthetic polymers have characteristic ratios similar to the two studied copolymers here.^21^

**Figure 6:**
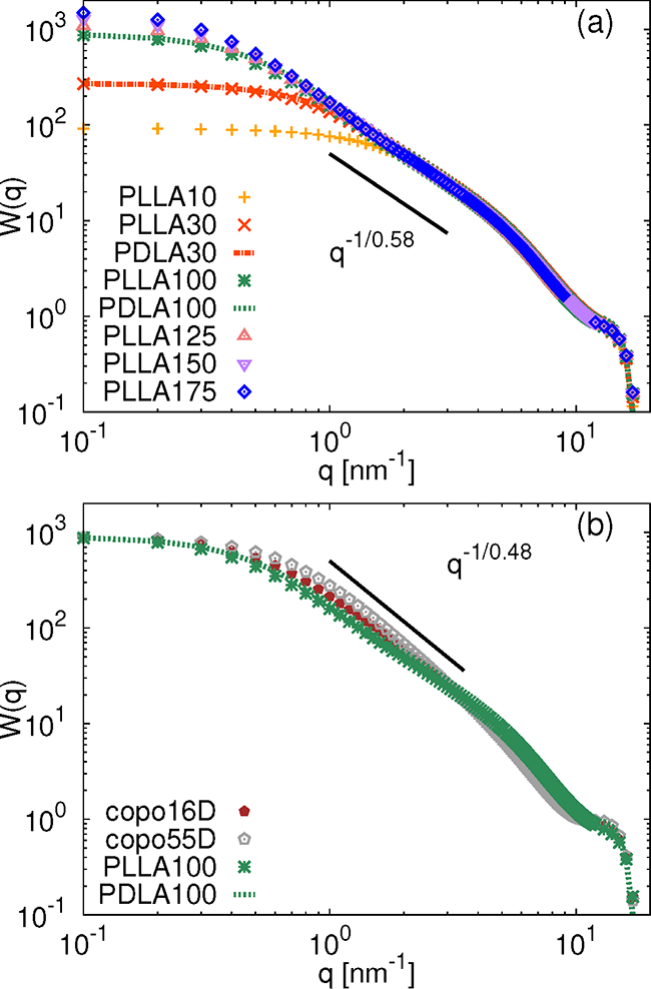
Form factor for (a) homopolymer and (b) copolymer systems. The solid black lines are guides for the eye for possible power law dependencies in the given *q* region.

As shown in Figure S7a in the Supporting Information, the percentage of hydrogen bonds in our systems is low, as expected due to the presence of only two donors per chain, namely, the terminal carboxyl and hydroxyl groups, as well as due to the relatively high temperature of 500 K. Data in Figure 7a show that in the case of homopolymers, only about 15% of the hydrogen bonds are intramolecular, which is in line with the aforementioned behavior of linear chains capable of forming reversible bonds. For the copolymers, this percentage is clearly higher, around 20%, which may be related to their higher flexibility and the more spherical nature of the local domains. Comparing Figure S7b with Figure 7a, it is evident that most of these intramolecular hydrogen bonds (between 60 and 70%) are formed at the chain extremes and cause an effect similar to backbiting (see inset of Figure S7b). It should be noted that the structure formed by such a bond resembles a seven-membered ring, and that it was found recently that the bond in the seven-membered ring is the strongest intramolecular H-bond in linear aminoalcohols,^40^ despite the fact that due to the entropy effects, the six-membered ring is the preferred conformation. These types of reversible bonds involving neighboring monomers are not effective in chain folding. On the other side, as the percentage of hydrogen bonds participating in backbiting is the same for copolymers and homopolymers, the difference for those two types of systems seen in Figure 7a must originate in a higher probability of finding hydrogen bonds connecting internal atoms in the copolymers.

**Figure 7:**
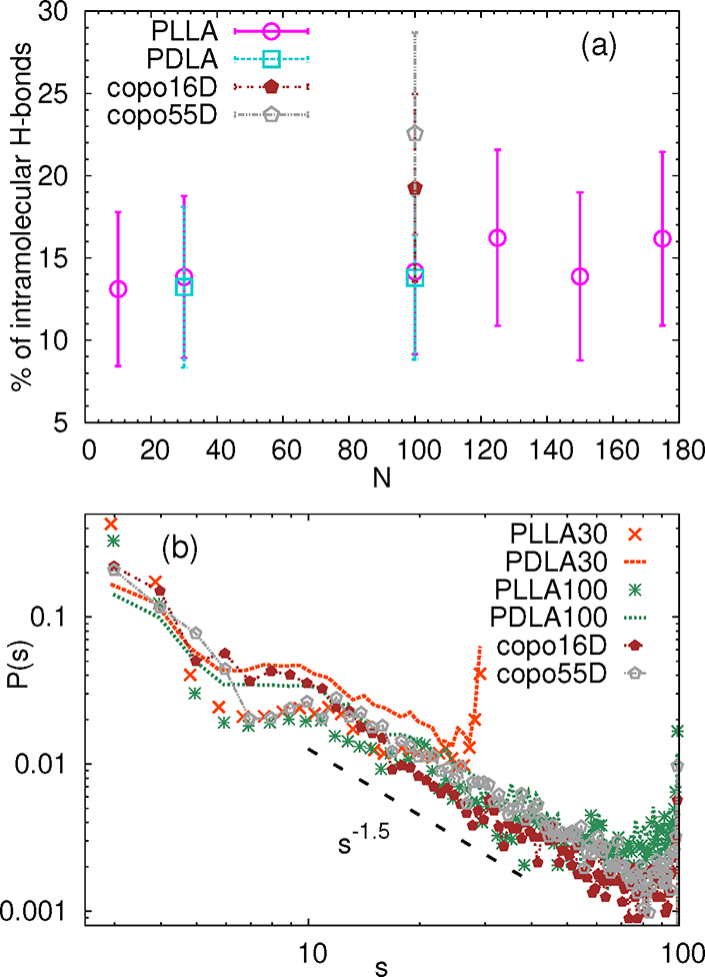
(a) Percentage of the intramolecular hydrogen bonds as a function of *N* for all simulated systems. (b) Distribution of chemical distances *s* between the monomers forming intramolecular hydrogen bonds. The dashed line is a guide for the eye and indicates a possible power law.

Excluding the hydrogen bonds involved in backbiting, in Figure 7b, we examine the distribution of the chemical distances *s* between the monomers forming an intramolecular hydrogen bond, i.e., *s* = |*i* – *j*|, where *i* and *j* are the indices of the monomers, with the aim of identifying the bonds that may cause the changes in local conformation. In the case of PLLA, the distributions exhibit a clear local minimum, followed by a local maximum around *s* ≈ 10. A barely noticeable minimum in the range *s* < 8 is detected for PDLA systems. This feature, namely, an appearance of a local minimum and maximum at short contour distances, was reported for single-chain nanoparticles with intramolecular irreversible bonds with a semiflexible character.^20^ More specifically, it was shown in ref 20 that there is no local minimum in the distribution of *s* in single-chain nanoparticles prepared from fully flexible linear chains (precursors), while the higher the stiffness of the precursor chain, the more pronounced minimum appears in the distribution. Despite the dissimilarities of our fully atomistic model and the coarse-grained model in ref 20, these reported features are in qualitative agreement with our results on the slightly more flexible character of PDLA than PLLA (see Figure 3). Concerning the copolymers, the data for copo55D closely follow the distributions for PLLA, and those for copo16D match the behavior of the PDLA chains. In addition, the intermediate decay of the distribution for *s* > 10 follows a power law with an exponent very close to −1.5, which is consistent with the behavior reported for single-chain nanoparticles.^20^ However, in contrast to the single-chain nanoparticles, the distributions for our PLA systems show an upturn for the distances equal to the chain length, which is a clear indication of a predominant tendency to form hydrogen bonds between the opposite terminal monomers. As also discussed in ref 20, the collapsing of the molecules is promoted by the formation of long “loops”, i.e., bonds at large distances *s*. Since bonds in this study are reversible and the probability of finding bonds separated by a long distance is relatively small, with the exception of the formation of the bond between the opposite terminal monomers, we anticipate that hydrogen bonding, which becomes more frequent at lower temperatures, may cause some local compactness of the chains similar to the sparse domains of the single-chain nanoparticles, combined with the overall globular character coming from the connection between the two extremes.

## Conclusions

Following the progress in implementing more sustainable methods in the polymer industry, we investigated in detail the structural properties of one of the most popular biobased polymers, poly­(lactic acid) (PLA). We employed atomistic molecular dynamics simulations to model different stereoisomers of PLA, namely, PDLA, PLLA, and two random copolymers with a distinct percentage of the D content. In addition, PLLA chains of multiple molecular weights were examined to highlight the effect of chain length.

The two studied homopolymers, PLLA and PDLA, exhibit very similar overall structural properties, represented by the asphericity and the form factor. Some minor differences in the radius of gyration, packing length, and the distribution of the distances between the intramolecular hydrogen bonds can be attributed to the slightly higher flexibility of the PDLA chains with respect to the PLLA homopolymers. Interestingly, and despite the described similarities, clear differences in the geometrical parameters related to the spatial orientation of adjacent monomers were detected. The behavior of both homopolymers reaches the Gaussian limit for chains containing more than 100 monomers, even though this limit seems slightly lower for PDLA chains and depends on the property under study.

The copolymer chains are more compact, more flexible, and consist of more spherical local domains than their homopolymer counterparts. We observed that the compactness of these molecules goes hand in hand with the higher percentage of intramolecular hydrogen bonds. It should be noted that these two features are intercorrelated; higher flexibility means higher conformational freedom and thus a higher probability of an ideal arrangement for the formation of an intramolecular hydrogen bond. At the same time, the formation of intramolecular bonds favors local compactness and the creation of local domains, which in effect leads to lower values of *c*_*∞*_. In addition, the spatial arrangement of the adjacent monomers in these systems seems to be affected by the local environment, i.e., by the presence of both types of monomers, L and D. This effect is usually not taken into account in theoretical models based on a single-chain configuration and may significantly affect the local dynamical properties of these materials. The dynamical as well as the rheological properties, which are sensitive to the local stereochemistry and are directly connected to the material performance during additive manufacturing, will be the topic of future study.

Overall, the structural properties of amorphous PLA chains in the melt at a temperature above the melting temperature closely resemble some features of commonly known synthetic polymers of comparable polymer length. In addition, we found intriguing evidence of some similarities in the distribution of intramolecular hydrogen bonds and in the fractal dimension with studies dealing with multiple types of single-chain nanoparticles. As these soft objects, composed of generic linear chains capable of reversible and/or irreversible intramolecular bonding, are a model system for intrinsically disordered proteins,^41,42^ and their peculiar intramolecular structure can be directly linked to their dynamical properties,^43^ we speculate that they may also serve as a convenient model system for flexible biobased polymers with hydrogen bonds. Nevertheless, as the effect of hydrogen bonding is particularly important at temperatures at which PLLA and PDLA also contain crystalline regions not considered here, we leave this possible likeness open for further study.

## Supplementary Material





## Notes

Additional information to the used model, additional evidence on different flexibility of studied stereoisomers, comparison of the calculated packing length and the asphericity distributions with the published data, geometrical parameters defining the n−π* interaction, and additional data on the analysis of hydrogen bonds in the systems (PDF)
Gromacs input files for PDLA30 and for the two studied copolymers; input files for the PLLA chains can be found elsewhere^44^ (ZIP)

## References

[1] European Commission . Strategic Plan 2020–2024. 2020. https://commission.europa.eu/publications/strategic-plan-2020-2024-research-and-innovation_en (accessed May 2025).

[2] Hegab H., Khanna N., Monib N., Salem A. (2023). Design for sustainable
additive manufacturing: A review. Sustainable
Materials and Technologies.

[3] Muringayil
Joseph T., Kallingal A., Suresh A., Kar Mahapatra D., Hasanin M., Haponiuk J., Thomas S. (2023). 3D printing of polylactic
acid: recent advances and opportunities. Int.
J. Adv. Manuf. Technol..

[4] Taib N.-A., Rahman M., Huda D., Kuok K., Hamdan S., Bakri M. K., Julaihi M., Khan A. (2022). A Review on Poly Lactic
Acid (PLA) as A Biodegradable Polymer. Polym.
Bull..

[5] Bikiaris N., Koumentakou I., Samiotaki C., Meimaroglou D., Varytimidou D., Karatza A., Kalantzis Z., Roussou M., Bikiaris R., Papageorgiou G. (2023). Recent Advances
in the Investigation of Poly­(lactic acid) (PLA) Nanocomposites: Incorporation
of Various Nanofillers and their Properties and Applications. Polymers.

[6] Jamshidian M., Tehrany E. A., Imran M., Jacquot M., Desobry S. (2010). Poly-Lactic
Acid: Production, Applications, Nanocomposites, and Release Studies. Comprehensive Reviews in Food Science and Food Safety.

[7] Tonelli A. E., Flory P. J. (1969). The Configurational
Statistics of Random Poly­(lactic
acid) Chains. I. Experimental Results. Macromolecules.

[8] Newberry R. W., Raines R. T. (2013). n→π∗
interactions in poly­(lactic
acid) suggest a role in protein folding. Chem.
Commun..

[9] Suzuki Y., Watanabe T., Kosugi H., Ueda K., Kikuchi M., Narumi A., Kawaguchi S. (2020). Chain Conformation
of Poly­(d-lactide)
in Tetrahydrofuran by Static Light Scattering, Small-Angle X-ray Scattering,
and Intrinsic Viscosity. Macromolecules.

[10] Sasanuma Y., Touge D. (2014). Configurational statistics
of poly­(L-lactide) and poly­(DL-lactide)
chains. Polymer.

[11] Brant D. A., Tonelli A. E., Flory P. J. (1969). The Configurational
Statistics of
Random Poly­(lactic acid) Chains. II. Theory. Macromolecules.

[12] Michell R. M., Ladelta V., Da Silva E., Müller A. J., Hadjichristidis N. (2023). Poly­(lactic acid) stereocomplexes based molecular architectures:
Synthesis and crystallization. Prog. Polym.
Sci..

[13] Glova A. D., Falkovich S. G., Dmitrienko D. I., Lyulin A. V., Larin S. V., Nazarychev V. M., Karttunen M., Lyulin S. V. (2018). Scale-Dependent
Miscibility of Polylactide and Polyhydroxybutyrate: Molecular Dynamics
Simulations. Macromolecules.

[14] Christofi E., Bačová P., Harmandaris V. A. (2024). Physics-Informed Deep Learning Approach
for Reintroducing Atomic Detail in Coarse-Grained Configurations of
Multiple Poly­(lactic acid) Stereoisomers. J.
Chem. Inf. Model..

[15] Guseva D. V., Glagolev M. K., Lazutin A. A., Vasilevskaya V. V. (2023). Revealing
Structural and Physical Properties of Polylactide: What Simulation
Can Do beyond the Experimental Methods. Polym.
Rev..

[16] Guseva D., Lazutin A., Vasilevskaya V. (2021). Atomistic
simulation of poly (lactic
acid) of different regioregularity. Polymer.

[17] Song J., Gomes G.-N., Gradinaru C. C., Chan H. S. (2015). An Adequate Account
of Excluded Volume Is Necessary To Infer Compactness and Asphericity
of Disordered Proteins by Förster Resonance Energy Transfer. J. Phys. Chem. B.

[18] Rawat N., Biswas P. (2009). Size, shape, and flexibility of proteins
and DNA. J. Chem. Phys..

[19] Rawat N., Biswas P. (2011). Shape, flexibility and packing of proteins and nucleic
acids in complexes. Phys. Chem. Chem. Phys..

[20] Moreno A. J., Bačová P., Lo Verso F. L., Arbe A., Colmenero J., Pomposo J. A. (2017). Effect of chain stiffness on the structure of single-chain
polymer nanoparticles. J. Phys.: Condens. Matter.

[21] Gkolfi E., Bačová P., Harmandaris V. (2021). Size and Shape
Characteristics of
Polystyrene and Poly­(ethylene oxide) Star Polymer Melts Studied By
Atomistic Simulations. Macromol. Theory Simul..

[22] Paciolla M., Likos C. N., Moreno A. J. (2022). Validity
of Effective Potentials
in Crowded Solutions of Linear and Ring Polymers with Reversible Bonds. Macromolecules.

[23] Prasitnok K. (2016). A coarse-grained
model for polylactide: glass transition temperature and conformational
properties. Journal of Polymer Research.

[24] Glagolev M., Glova A., Mezhenskaia D., Falkovich S., Larin S., Vasilevskaya V., Lyulin S. (2018). Coarse-grained A-graft-B
model of poly­(lactic acid) for molecular dynamics simulations. J. Polym. Sci., Part B: Polym. Phys..

[25] Behbahani A. F., Schneider L., Rissanou A., Chazirakis A., Bačová P., Jana P. K., Li W., Doxastakis M., Polińska P., Burkhart C., Müller M., Harmandaris V. (2021). Dynamics and rheology of polymer melts via hierarchical
atomistic, coarse-grained, and slip-spring simulations. Macromolecules.

[26] Androsch, R. ; Schick, C. ; Di Lorenzo, M. L. Synthesis, Structure and Properties of Poly(lactic acid); Di Lorenzo, M. L. ; Androsch, R. , Eds.; Springer International Publishing: Cham, 2018; pp 235–272.

[27] Abraham M. J., Murtola T., Schulz R., Páll S., Smith J. C., Hess B., Lindahl E. (2015). GROMACS: High
performance
molecular simulations through multi-level parallelism from laptops
to supercomputers. SoftwareX.

[28] McAliley J. H., Bruce D. A. (2011). Development of Force
Field Parameters for Molecular
Simulation of Polylactide. J. Chem. Theory Comput..

[29] Ryckaert J.-P., Bellemans A. (1975). Molecular
dynamics of liquid n-butane near its boiling
point. Chem. Phys. Lett..

[30] Hess B., Bekker H., Berendsen H. J. C., Fraaije J. G. E. M. (1997). LINCS: A linear
constraint solver for molecular simulations. J. Comput. Chem..

[31] Bačová, P. PLA Analysis Tools. 2023. https://github.com/pbacova/PLA_analysis_tools.git (accessed May 2025).

[32] Rubinstein, M. ; Colby, R. H. Polymer physics; Oxford University Press, 2003.

[33] Glova A. D., Falkovich S. G., Larin S. V., Mezhenskaia D. A., Lukasheva N. V., Nazarychev V. M., Tolmachev D. A., Mercurieva A. A., Kenny J. M., Lyulin S. V. (2016). Poly­(lactic acid)-based
nanocomposites filled with cellulose nanocrystals with modified surface:
all-atom molecular dynamics simulations. Polym.
Int..

[34] Dorgan J.
R., Janzen J., Knauss D. M., Hait S. B., Limoges B. R., Hutchinson M. H. (2005). Fundamental
solution and single-chain properties of
polylactides. J. Polym. Sci., Part B: Polym.
Phys..

[35] Othman, N. Rheology and processing of poly­(lactides) and their enantiomeric copolymers and blends. Ph.D. thesis, University of British Columbia, 2012.

[36] Unidad H.
J., Goad M. A., Bras A. R., Zamponi M., Faust R., Allgaier J., Pyckhout-Hintzen W., Wischnewski A., Richter D., Fetters L. J. (2015). Consequences
of Increasing Packing
Length on the Dynamics of Polymer Melts. Macromolecules.

[37] Everaers R., Karimi-Varzaneh H. A., Fleck F., Hojdis N., Svaneborg C. (2020). Kremer-Grest
Models for Commodity Polymer Melts: Linking Theory, Experiment, and
Simulation at the Kuhn Scale. Macromolecules.

[38] Formanek M., Rovigatti L., Zaccarelli E., Sciortino F., Moreno A. J. (2021). Gel Formation in
Reversibly Cross-Linking Polymers. Macromolecules.

[39] Bačová P., Li W., Behbahani A. F., Burkhart C., Polińska P., Doxastakis M., Harmandaris V. (2021). Coupling between Polymer Conformations
and Dynamics Near Amorphous Silica Surfaces: A Direct Insight from
Atomistic Simulations. Nanomaterials.

[40] Li Y., Yang X., Yu Y., Zhou X., Zhang R., Sun J., Liu S. (2023). Dependence
of Intramolecular Hydrogen Bond on Conformational
Flexibility in Linear Aminoalcohols. J. Phys.
Chem. A.

[41] Moreno A. J., Lo Verso F., Arbe A., Pomposo J. A., Colmenero J. (2016). Concentrated
Solutions of Single-Chain Nanoparticles: A Simple Model for Intrinsically
Disordered Proteins under Crowding Conditions. J. Phys. Chem. Lett..

[42] Huo M., Wang N., Fang T., Sun M., Wei Y., Yuan J. (2015). Single-chain polymer nanoparticles: Mimic the proteins. Polymer.

[43] Mei B., Moreno A. J., Schweizer K. S. (2024). Unified Understanding of the Structure,
Thermodynamics, and Diffusion of Single-Chain Nanoparticle Fluids. ACS Nano.

[44] Christofi, E. PLA Backmapping. 2023. https://github.com/SimEA-ERA/PLA-BackMap-CG (accessed May 2025).

