# Supplementary material for: Computational Investigation of the Structural Properties of Poly(lactic acid) and Its Stereoisomers: Shape, Size, and Flexibility
Source: Macromolecules. 2025 Jul 15;58(16):8572–80. doi: 10.1021/acs.macromol.5c00918 (PMC12392737; doi:10.1021/acs.macromol.5c00918)
Supplement: Supplementary file 1 [file ma5c00918_si_001.pdf]

Supporting Information: Computational investigation of structural properties of poly(lactic acid) and its stereoisomers: shape, size and flexibility

Petra Bačová,[*,†] Vagelis Harmandaris,[‡,¶,§] and Sergio I. Molina[†]

†*Departamento de Ciencia de los Materiales e Ingeniería Metalúrgica y Química Inorgánica, Facultad de Ciencias, IMEYMAT, Campus Universitario Río San Pedro s/n., Puerto Real, Cádiz 11510, Spain*

‡*Computation-based Science and Technology Research Center, The Cyprus Institute, 20 Constantinou Kavafi Str., Nicosia 2121, Cyprus*

¶*Institute of Applied and Computational Mathematics (IACM), Foundation for Research and Technology Hellas (FORTH), GR-70013 Heraklion, Crete, Greece*

§*Department of Mathematics and Applied Mathematics, University of Crete, GR-71409 Heraklion, Crete, Greece*

E-mail: petra.bacova@uca.es

Adaptation of the force field to the copolymer systems

The PLAFF3 force field[S1] was developed to reproduce experimentally resolved crystal structure conformations, melt density, volume expansivity, and the glass transition temperature.

To achieve these objectives, the dihedral angle potential was enriched with CMAP dihedral cross terms and one of the backbone dihedrals was modelled with the tabulated form, adjusted for the Gromacs simulation package.[S2] The tabulated dihedral for both, L and D form of PLA is shown in Fig. S1. There are two visible features of this approach: (a) the tabulated potentials for both stereoisomers have similar shape, but are a mirror image of each other, (b) the potential is approximated by a set of points, i.e., it is discretized. To simplify and unify the model in order to be able to simulate copolymer structures, we substituted the tabulated potential for both types of PLA monomers with the functional form of Ryckaert-Bellemans function:[S3]

$$V_{rb}(\phi_{ijkl}) = \sum_{m=0}^{5} C_m(\cos(\psi))^m, \tag{1}$$

where C_m represent the main parameters. The chosen functional form, which represents the best fit to the tabulated potentials reported in ref.[S1] is shown in Fig. S1. The values of the parameters were adjusted to $C_0 = 65.9339$ kJ/mol, $C_1 = 8.48321$ kJ/mol, $C_2 = -75.2043$ kJ/mol and $C_3 = C_4 = C_5 = 0$ kJ/mol. As also mentioned in our previous

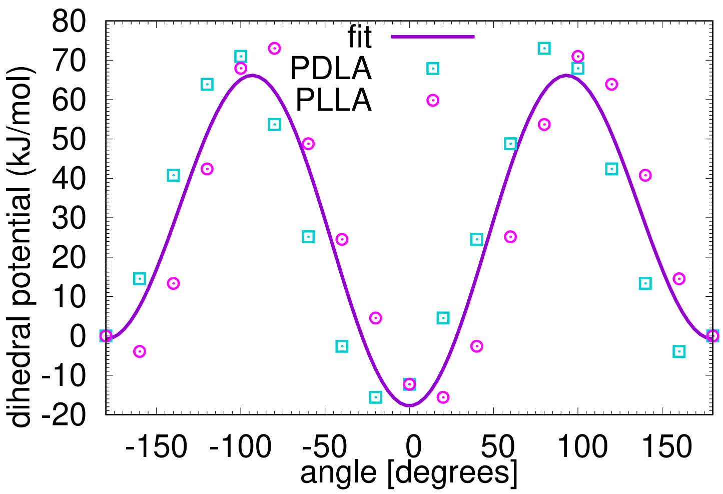

Figure S1: Backbone dihedral potential for the PLLA and PDLA homopolymers according to ref.[S1](points) and after applying the approximation by the Ryckaert-Bellemans function (line, see the text for the functional form).

work,[S4] a simultaneous usage of two tabulated potentials for the dihedral potential is not facilitated in Gromacs simulation package and therefore, the implementation of its functional form as explained in eq. 1 was necessary to avoid the simplification used in our previous work.[S5] In addition, it is important to stress out that two types of CMAP dihedrals must be implemented in the case of the copolymer structure and therefore, two types of chiral centers (L and D) must be distinguished in the atom types list. The whole procedure can be seen in the input files included as a part of the Supporting Information.

In order to quantify possible effect of substituting the tabulated potential with the functional form, we simulated the homopolymers employing both options. The radius of gyration obtained by both employed methods, i.e., the tabulated or the functional form, are summarized in Table S1. In the case of PLLA, the maximum deviation for both models is 3.4% for PLLA30. For PDLA polymers, the average values of R_g are lower in the simulations with the functional form in contrast to those with the tabulated potential, with the maximum deviation of 6.6% in PDLA100. Minor differences were found in other structural properties presented in this work when comparing the two forms of dihedral potential, the majority of them, however, is negligible within the obtained accuracy.

It is important to stress out that the conclusions presented in the main manuscript related to the comparison of PLLA and PDLA are valid for both types of simulations with the employed dihedral potential, namely, the Gaussian character of the chain found for $N > 100$, the slightly lower C_∞ for PDLA than for PLLA, the shift of the maximum in the asphericity distribution towards lower values for PLLA chains with higher molecular weights, the seemingly identical form factors for PLLA and and PDLA. In addition, the positions of the maxima in the probability distribution functions of the distance d (see Fig. S6 below) are identical for the two types of simulations of homopolymers. The largest quantitative differences were detected in the analysis of the hydrogen bonds. More specifically, in the case of the PDLA chains simulated with the tabulated potential, the chains showed a much higher tendency to form intramolecular bonds than their PLLA analogues (data not shown),

particularly at the extremes of the chain. On the other hand, in the simulations with the functional form a more consistent data set was found and the trend was in accordance with the tendencies observed in the other structural properties (see Fig. 6(a) in the main manuscript). Having in mind all aforementioned findings and the fact that the simulations with the functional form of the dihedral potential represent the only set of homopolymers which can be fairy compared with the copolymer systems, in what follows we report only the data for the simulations with the dihedral potential expressed with the functional form shown in eq. 1.

Table S1: Comparison of the radius of gyration obtained in this work by employing the functional form for the backbone dihedral, R_g, and the one obtained by employing the tabulated form of the dihedral potential, as suggested in ref.,[S1] R_g^r.

system label	R_g [nm]	R_g^r [nm]
PLLA10	0.758±0.001	0.781±0.001
PLLA30	1.56±0.02	1.615±0.006
PDLA30	1.47±0.02	1.56±0.02
PLLA100	3.30±0.03	3.31±0.03
PDLA100	2.96±0.01	3.17±0.03
PLLA125	3.59±0.03	3.60±0.04
PLLA150	3.88±0.02	3.86±0.01
PLLA175	4.20±0.02	4.10±0.07
copo16D	2.61±0.05	x
copo55D	2.218±0.005	x

Other evidence on dissimilarities in flexibility of different stereoisomers

As shown in Fig. 3 and discussed in the main manuscript, the studied stereoisomers manifest some differences in flexibility. Since the persistence length, l_p, is the most common indicator of the chain flexibility, we briefly comment on two ways that could lead to the persistence

length estimation. It is important to stress that the exact estimation of l_p is beyond the scope of this work and that in the case of the atomistic models the direct connection of the measured variables to the theoretical models may be a point of discussion.

In terms of interpreting the experimental data and in many cases also the data from the simulations of biomolecules, the worm-like chain (WLC) model is the most popular choice. It describes well polymers with relatively high l_p, as it assumes small values of the bond angle Θ and the main contribution to the flexibility being the chain contour fluctuations.[S6] The model offers an expression for the relation of the end-to-end distance R_e^2 to the contour length $R_{\max}$:

$$\left\langle R_e^2 \right\rangle = 2l_p \left\langle R_{\max} \right\rangle (1 - \frac{l_p}{\left\langle R_{\max} \right\rangle}[1 - \exp(- \left\langle R_{\max} \right\rangle / l_p)]). \tag{2}$$

In the case of the WLC model, $R_{\max} \cong nl_b$, where n is the number of bonds along the backbone and l_b is their average bond length. Since in our systems there are different types of bonds along the backbone, we calculated the contour length as $R_{\max} = \sum_t n^t l_b^t$, where n^t is the number and l_b^t the length of the specific bond, e.g., the bond between the ester oxygen and the chiral carbon (see e.g., Fig. S3(a)). The dependency of R_e^2 on $R_{\max}$ is shown in Fig. S2.

Having in mind that the flexibility of atomistic models such as PLA studied here is mostly given by the internal rotation around the backbone bonds, the WLC model is not a suitable choice for interpreting the data obtained in this work. Nevertheless, due to the popularity of the WLC model and for the sake of comparison with the studies which made use of this model, we applied the eq. 2 to adjust our data, keeping l_p as the fitting parameter. The obtained values of l_p are 0.76 nm for PLLA homopolymers and 0.64 nm for PDLA homopolymers. Note that only 2 points were used in the estimation of l_p of PDLA and that the data for the shortest PLLA chain, PLLA10, had to be excluded to be able to adjust the model. The obtained values compare well with the previously published simulation and experimental data.[S7]

Another way of quantifying the flexibility of the chain is by monitoring the correlation

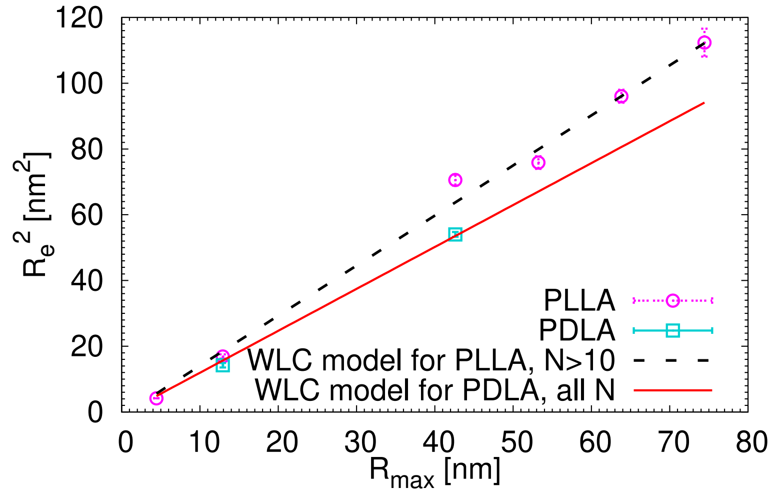

Figure S2: End-to-end distance $\langle R_e^2 \rangle$ as a function of the maximum contour length $R_{\max}$ for all studied homopolymers. The dash line represents the fit to the worm-like chain model (WLC) for PLLA systems, excluding the shortest chain with $N = 10$. The solid line is the fit of the PDLA data to the WLC model.

function of the vectors along the backbone:[S6]

$$C(s) = \langle \cos \Theta(s) \rangle = \frac{\vec{v_i}.\vec{v_{i+s}}}{|\vec{v_i}||\vec{v_{i+s}}|} \tag{3}$$

where $\vec{v_i}$ represents a vector connecting different entities along the backbone (see below) and s is the difference between their indices. This correlation function has been mostly studied in coarse-grained models, where $\vec{v_i}$ is defined as a bond vector. Due to different types of bonds in our models, we tested 3 definitions of this vector: $v1$, which connects the ester oxygen with the chiral center, $v2$, which connects the consecutive ester oxygens and $v3$ which connects the centers of masses of the consecutive monomers. The three vectors are illustrated in Fig. S3(a).

As the distance s between the vectors increases, the correlation function decreases, with l_p being the measure of the fast decay of the structural decorrelation:[S6,S8]

$$C(s) = \exp\left(-s l_b / l_p\right). \tag{4}$$

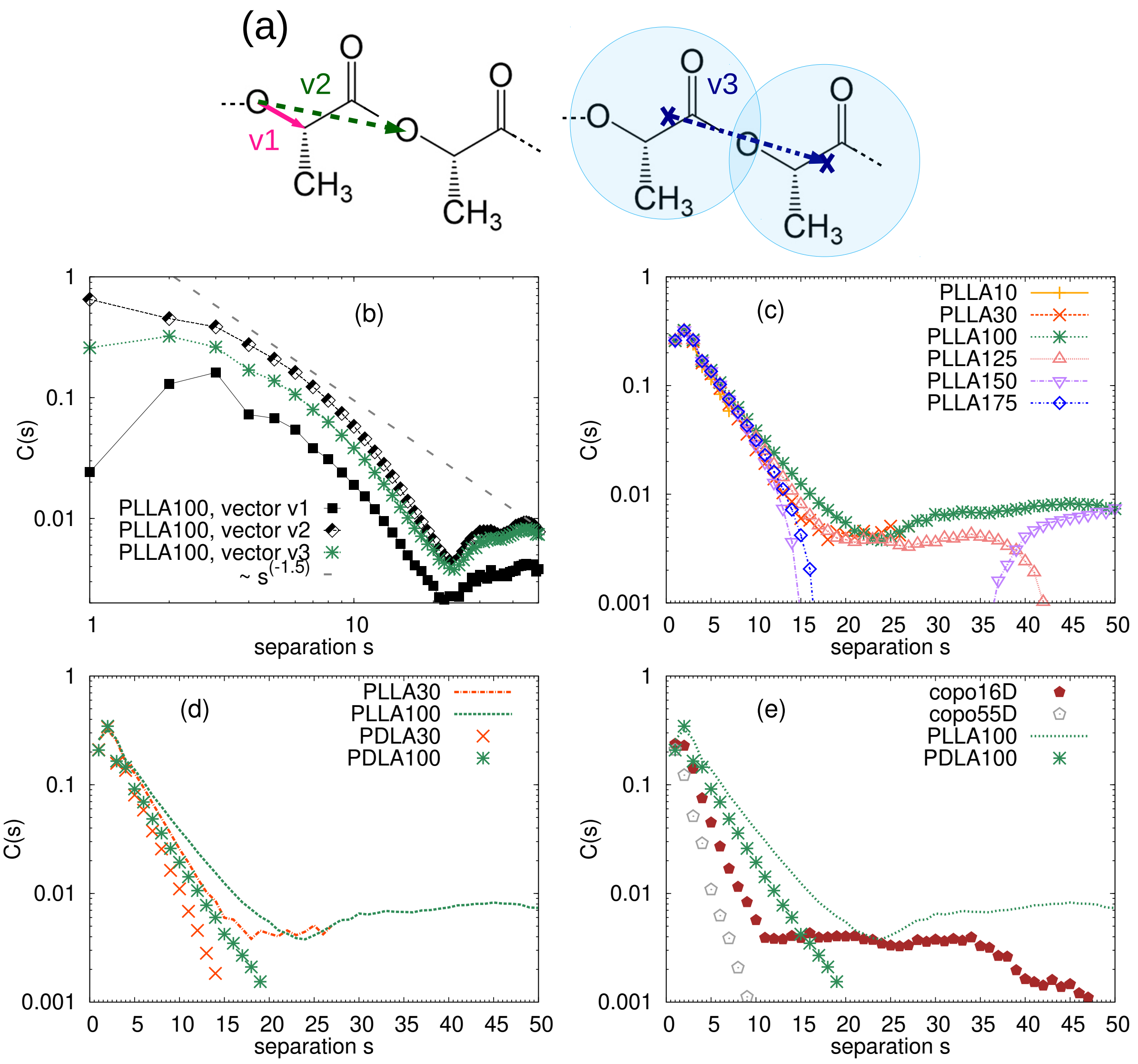

Figure S3: Correlation functions of the vectors along the backbone. (a) Schematic illustration of the vectors. (b) Correlation function $C(s)$ for PLLA100 for vectors shown in (a). The dashed line indicates the power law with the exponent of -1.5. (c) $C(s)$ as a function of s for the vector $v3$ for PLLA chains of different molecular weight. (d) $C(s)$ as a function of s for the vector $v3$ for PLLA and PDLA homopolymers. (e) $C(s)$ as a function of s for the vector $v3$ for different stereoisomers with the chain length of $N = 100$. Note that some values of $C(s)$ are negative and therefore are not visible in a logarithmic representation.

In the case of dense systems, such as melt studied here, it has been suggested that $C(s)$ may exhibit a power law decay:[S8–S10]

$$C(s) \sim s^{-1.5} \tag{5}$$

As seen from comparing Fig. S3(b) and Fig. S3(c), our data seem to be in line with eq. 4 rather than with eq. 5. The exponential decay in melt was also observed for the atomistic models of polyethylene.[S11] In addition, our data for different types of vectors in Fig. S3(b) show the same tendency as the one reported in ref.[S11] Namely, comparing data for $v1$ and $v2$, a very similar decay of $C(s)$ can be observed, indicating the same l_p as also explained in ref.,[S11] however, with $C(s)$ for $v2$ being systematically over the $C(s)$ for $v1$, since $v2$ would correspond to longer separation in terms of the actual distance. Note that in ref.[S11] the different vectors corresponded to different coarse-graining levels, while here we probe different vectors connecting atoms along the chain. In order to be consistent with the studies using the coarse-grained representation, we chose $v3$ for the data shown in Fig. S3(c,d,e). The correlation functions for different stereoisomers corroborate the observations made in the main manuscript: the PDLA homopolymers are slightly more flexible than their PLLA analogues and the higher percentage of D-content in the copolymer leads to higher flexibility.

Comparing packing length with the published data

In Fig. S4 the packing length p calculated from our simulation data is compared to the data obtained from the literature.[S12] Note that in ref.[S12] the tacticity of polystyrene (PS) is not reported, however, in ref.[S13] the values of packing length for PS vary from 0.392 for atactic and 0.408 for isotactic PS. It is also important to stress out that the values for common synthetic polymers presented in Fig. S4 may slightly vary depending on the source of experimental data, temperature etc. (e.g., the difference in values of p for polyethylene in ref.[S13] and in ref.[S12] is around 20%).

Concerning the effect of the local packing on the macroscopic properties of these polymers, such as viscoelastic behavior, the packing length is closely connected to the entanglement molecular weight M_e through the following equation:[S12]

$$M_e = n_t^2 \rho \mathrm{N}_A p^3 \tag{6}$$

where n_t is a weakly temperature-dependent dimensionless number with a value of approximately 21.[S12] Having in mind the direct proportionality of p and M_e, it might be expected that the difference in p for the copolymer and homopolymer systems in Fig. S4 would lead to different viscoelastic behavior. This phenomenon is beyond the scope of this work, however, it was discussed for instance in ref.,[S14] where it was also pointed out that the hompolymer samples are semi-crystalline while the random copolymers are amorphous.

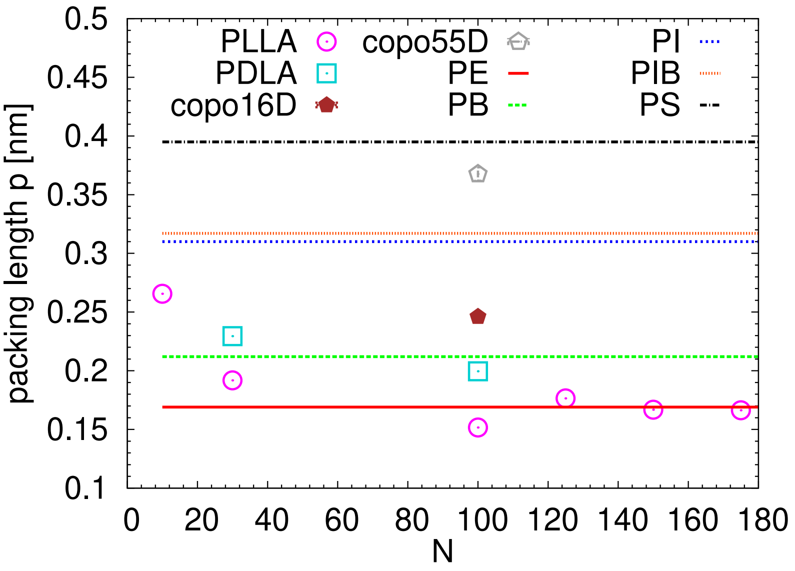

Figure S4: Packing length as a function of the number of monomers per chain. The horizontal lines are data published in ref.[S12] The error bars are of the order of the point size and have been calculated according to the error propagation theory using the data from Table 1 in the main manuscript.

Comparing asphericity with the published data

In Fig. S5 we compare the distribution of the asphericity parameter for the longest studied chain, PLLA175, with the data reported in the literature. In Fig. S5(a) we focus on the systems containing intramolecular reversible bonds, such as hydrogen bonds. Namely, we present the data for a double helical deoxyribonucleic acid (DNA), which is considered a semiflexible macromolecule,[S15] and the data for a generic coarse-grained (CG) model of linear chains containing 10% of the monomers capable of reversible bonding. These reactive

sites were distributed along the chain with a constant separation, however, the data for different types of distribution of the reactive sites are almost indistinguishable from each other for the given precision (see Fig. 2(c) in ref.[S16]).

Both systems from the literature, DNA and CG, represent isolated molecules, i.e., in infinite dilution, since our main focus in this comparison is the effect of the intramolecular bonds. The DNA in complexes with other molecules adapts more sherical shape, with a maximum in a distribution near 0.2.[S17] The asphericity of CG systems also decreases at higher concentrations, at which they form reversible gels.[S18]

The distribution of the asphericity parameter for CG systems closely follows the distribution for PLLA175. Namely, it is extended over the whole range from 0 to 1, with one main and a second apparent maximum. As also seen in Fig. S5(b) and discussed in refs.,[S19,S20] this type of distribution can be fitted to the sum of two Gaussian functions:

$$p(a_f) = B1\ \exp\left[-\left(\frac{a_f - b1}{s1}\right)^2\right] + B2\ \exp\left[-\left(\frac{a_f - b2}{s2}\right)^2\right] \tag{7}$$

where $B1, B2$ are weights, $b1, b2$ represent the positions of two maxima and $s1, s2$ are the respective dispersions. The existence of the two maxima indicates two conformational states of the chain, the collapsed one with the lower asphericity and the coil with the higher asphericity.

The distribution for DNA has the first maximum shifted to lower a value, due to the bias caused by chains consisting of only a few base pairs.[S15] Its main peak coincides with the maximum typical for the coil state, and therefore, despite having a complex biostructure and containing multiple intramolecular bonds, it shows some similarities with synthetically-prepared polymers in Fig. S5(b).

Concerning the synthetic polymers shown in Fig. S5(b), there is only a minor deviation among systems of different polymer type.

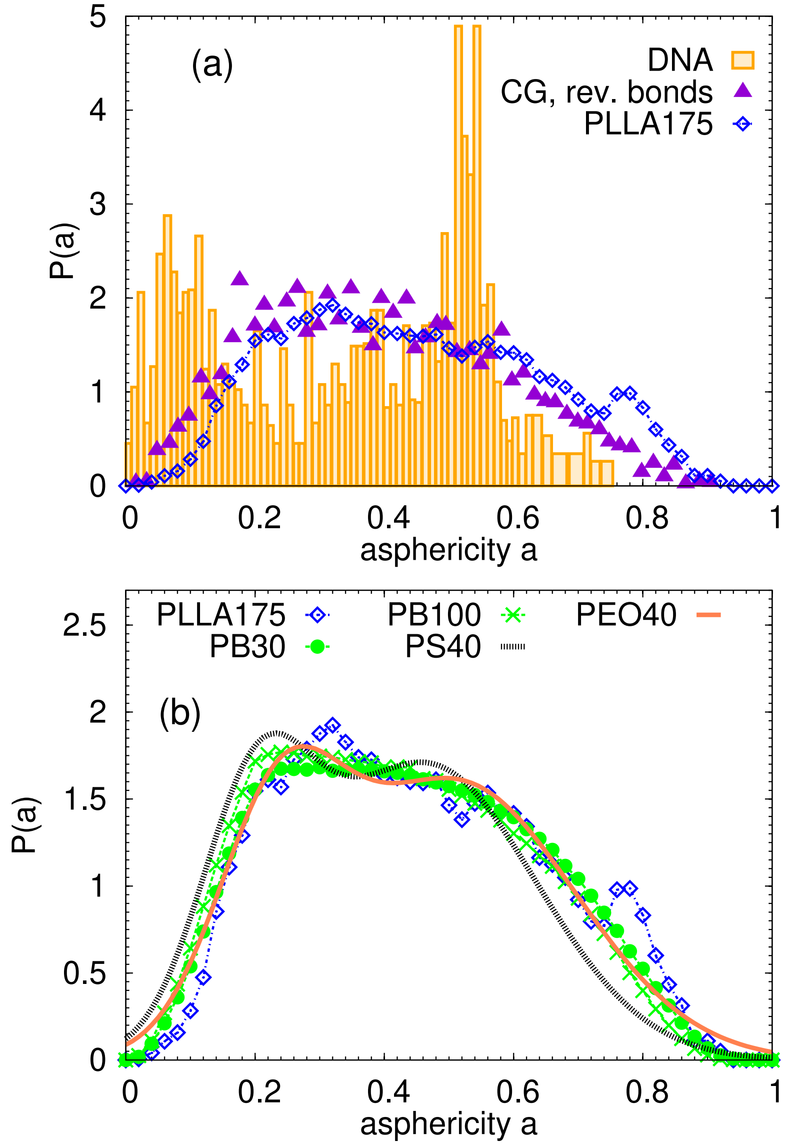

Figure S5: Distribution of the asphericity parameter for the macromolecules (a) forming reversible bonds and (b) some examples of synthetic polymers. The data for the DNA molecules were extracted from ref.,[S15] the data labelled as CG correspond to the simulation data for Kremer-Grest model for linear chain capable of forming reversible bonds.[S16] Both are single-molecule systems. The data in (b) represent the fits to eq. 7 reported in Supplementary Material in ref.[S20] for atactic polystyrene (PS) and poly(ethylene oxide) (PEO), both containing 40 monomers. The data labelled as PB in (b) are unpublished data from refs.[S21,S22] for *cis*-polybutadiene containing 30 and 100 monomers. All data in (b) are for systems in melt.

Geometrical parameters defining the n-π^* interaction

Even though the PLA systems studied here were simulated at the temperatures well-above the crystallization and glass transition temperature, we analyze the geometrical parameters defining the n-π^* interaction in the crystalline state of PLA to demonstrate the differences in the spatial orientation of the adjacent monomers among studied stereoisomers. As explained in ref.[S23] and also schematically shown in Fig. 5(a) of the main manuscript, these parameters consist of

- the distance d between the oxygen atom in the carbonyl group and the carbon atom in the next carbonyl group,
- the angle between the vector defining the distance d and the double C-O bond in the carbonyl
- the angle defining the pyramid conformation in the ester carbonyl.

The average values of these parameters reported for the α conformational isomer of PLA in the crystalline structure in ref.[S23] are plotted together with the distributions obtained from our simulations in Fig. S6 and in Fig. 5 of the main manuscript.

Concerning the actual values in Fig. S6(a,c) and in Fig. 5(b), the distances found in our simulations are larger than those in α-PLA. In α-helices in proteins, the prevailing distances are d$\leq$0.32 nm and $\theta \approx 100°$.[S24] This combination of geometrical parameters seems to be more in agreement with the data collected for PLLA than for PDLA, where the main peak for d is positioned around d$\approx$ 0.45 nm. Moreover, the main peak in the PLLA distribution of distances coincides with the Lennard-Jones parameter σ_{ij} obtained by applying geometrical mixing rules for the carbon-oxygen interaction of the two consecutive carbonyl groups (see the gray vertical line in Fig. 5(b)). Note that d$<\sigma_{ij}$ is one of the conditions determining the presence of an effective n-π^* interaction.

The distributions of distances and the angles are independent of the molecular weight within the achieved accuracy (data not shown). In addition the distributions of ψ for all

the systems are identical, representing a Gaussian function centered in 0, with a standard deviation of 4.1 (data not shown).

Concerning the differences among the studied stereoisomers, the data for homopolymers PLLA and PDLA exhibit clear differences in positions and amplitude of the maxima in Fig. 5(b) and Fig. S6(c), despite having very similar flexibility, shape and size (see the main manuscript). Since the distributions for the copolymer systems copy some features from both homopolymers, we tested if the distribution functions for the copolymer systems can be obtained by a linear combination of the homopolymers' distributions, e.g., for the distribution of distances the expression would be:

$$P_c(\mathrm{d}) = p_l P_{\mathrm{PLLA}}(\mathrm{d}) + p_d P_{\mathrm{PDLA}}(\mathrm{d}) \tag{8}$$

where p_l and p_d represent a fraction of every monomer type (L or D) in the copolymer. For instance, for the copolymer with 16% of the D content $p_d = 0.16$ and $p_d = 0.84$. These linear combinations are plotted together with the distributions calculated from the simulations in Fig. S6(b). The linear combinations capture the main features of the distributions, however, the agreement is worse for the copolymer with the higher content of D- monomer. Note that the linear combination is expected to be more suitable in the case of block copolymers, as it only accounts for the percentage of the distances between monomers of an identical stereochemistry. However, we cannot confirm this assumption and moreover, there might be a possible effect of the local environment. We attribute the observed discrepancy to the fact that the distances between consecutive L and D monomers are not considered in eq. 8. In order or examine also the effect of the environment, we plotted in Fig. S6(c) only the distributions for the LL and DD pairs in copolymers and compared them to the homopolymer case. In general, there are two main features in Fig. S6(c): i) the distributions for the LL and DD pairs in the copolymers are not identical with the distributions of d obtained for the corresponding homopolymers; ii) the agreement between the copolymers

and homopolymers is worse for the type of pair which is in minority in the given copolymer composition, i.e., for the DD pair in copo16D and for the LL pair in copo55D. In both cases in ii) the distributions show higher population of longer distances in homo-pairs belonging to the copolymers in comparison to their homopolymer analogues. These results suggest that the local packing in copolymer systems cannot be easily predicted from the knowledge of the local configuration of the homopolymer chains, as the spatial distribution of the adjacent monomers is affected by the local environment.

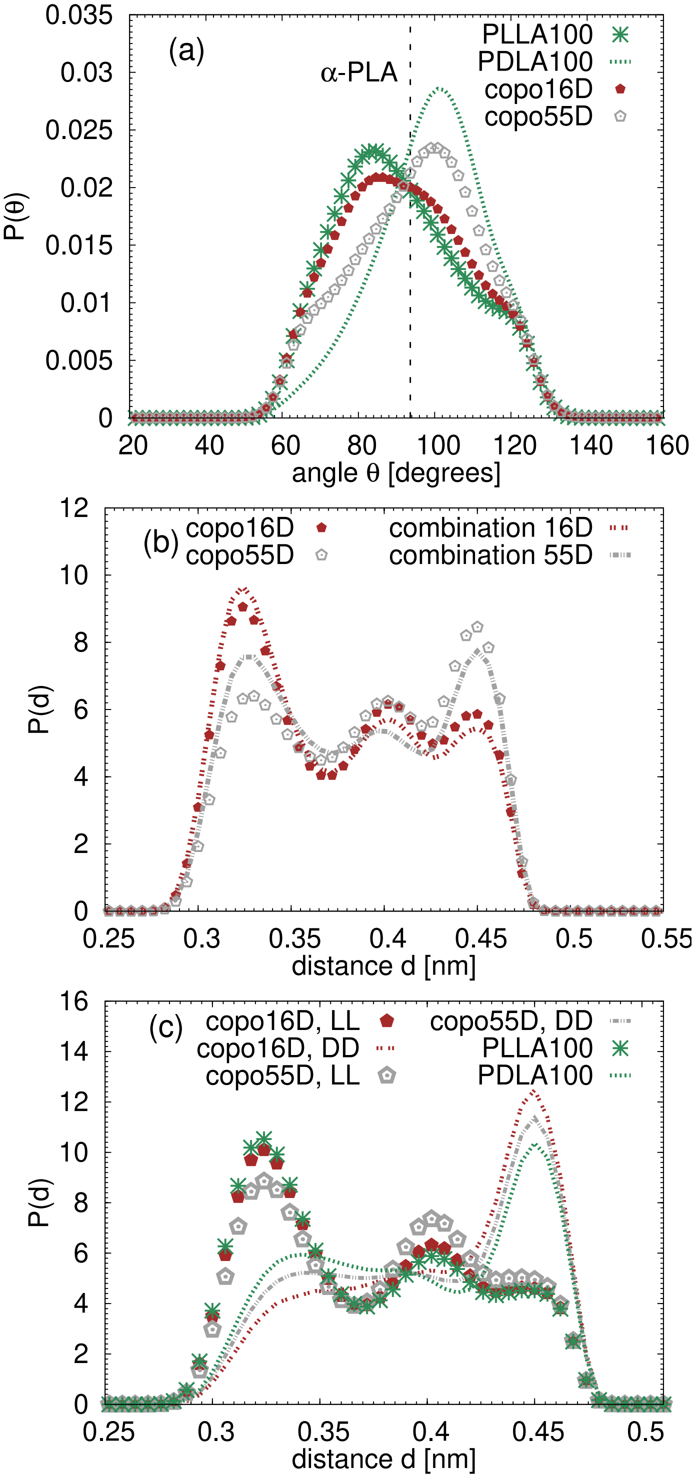

Figure S6: Geometrical parameters defining the n-π^* interaction. Probability distribution functions (a) of of the angle θ and (b,c) of the distance d for the studied systems. The dashed vertical line in (a) indicates the value reported for the α conformational isomer of PLA in ref.[S23] The lines in (b) correspond to the combination of the homopolymers' distributions (see the text). (c) The distributions of the distances d for the specific pairs of monomers in copolymers, namely LL and DD, in comparison to the homopolymers' distributions.

Presence of hydrogen bonds

As the hydrogen bonds govern the intramolecular structure in many bio-based molecules, we investigate the presence of the hydrogen bonds in our systems. Note that in the case of PLA, the terminal carboxyl and the hydroxyl groups are the only donors and therefore, the expected number of hydrogen bonds is low.

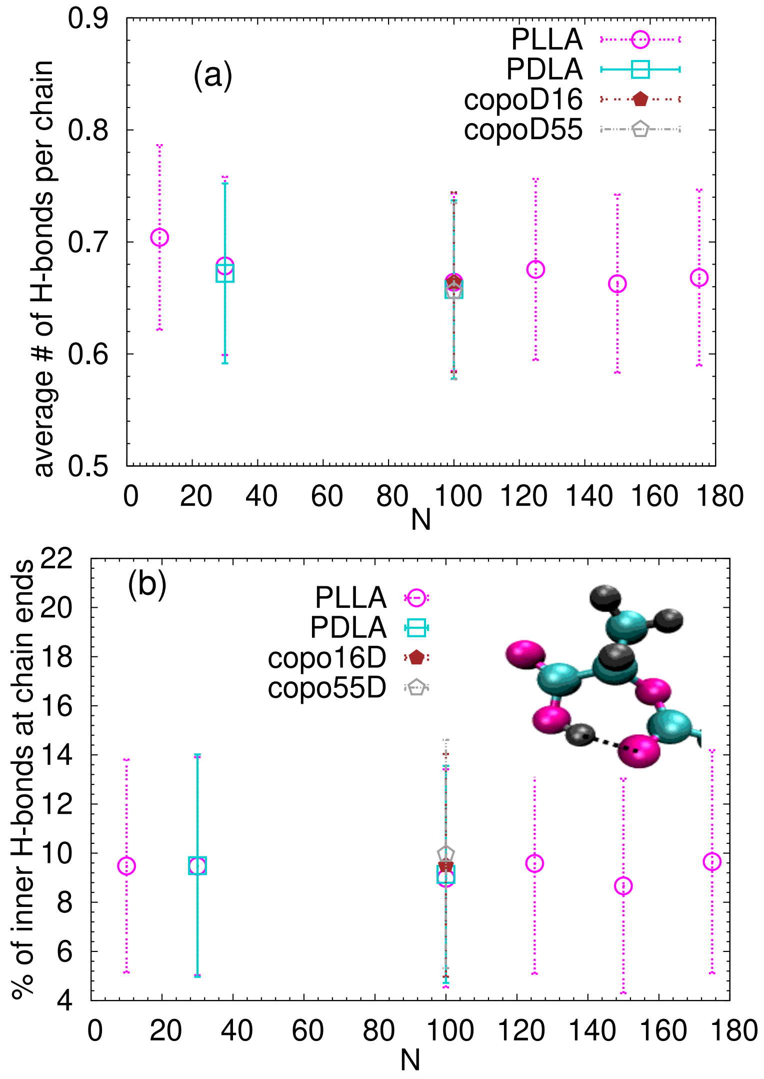

Figure S7: Hydrogen bonds at 500K: (a) Average number of hydrogen bonds per chain as a function of the chain length N. (b) Percentage of end-chain intramolecular hydrogen bonds as a function of N. The inset illustrates the formation of the bond between the terminal hydroxyl group and the nearest acceptor.

As shown in Fig. S7(a) this is indeed the case. The average number of hydrogen bonds per chain and per time frame is lower than 1, which means that in many cases only one of the terminal groups participates in the hydrogen bond formation or does not participate at all. The data indicate a relatively constant number of hydrogen bonds independent of the stereochemistry, which is expected as all chains contain the same terminal groups and thus the same number of donors.

In Fig. S7(b) it is shown that most of the intramolecular hydrogen bonds are those which cause the backbiting of the chain, i.e., the bonds which are formed at the extremes of the chains and therefore do not participate in the internal folding of the molecule. Such a situation is illustrated in the inset of Fig. S7(b).

References

(S1) McAliley, J. H.; Bruce, D. A. Development of Force Field Parameters for Molecular Simulation of Polylactide. *Journal of Chemical Theory and Computation* **2011**, *7*, 3756–3767, DOI: 10.1021/ct200251x.

(S2) Abraham, M. J.; Murtola, T.; Schulz, R.; Páll, S.; Smith, J. C.; Hess, B.; Lindahl, E. GROMACS: High performance molecular simulations through multi-level parallelism from laptops to supercomputers. *SoftwareX* **2015**, *1-2*, 19–25, DOI: https://doi.org/10.1016/j.softx.2015.06.001.

(S3) Lindahl, E.; Abraham, M. J.; Hess, B.; van der Spoel, D. GROMACS 2019 Manual. 2018; https://doi.org/10.5281/zenodo.2424486.

(S4) Christofi, E.; Bačová, P.; Harmandaris, V. A. Correction to "Physics-Informed Deep Learning Approach for Reintroducing Atomic Detail in Coarse-Grained Configurations of Multiple Poly(lactic acid) Stereoisomers". *Journal of Chemical Information and Modeling* **2024**, *64*, 6926–6926, DOI: 10.1021/acs.jcim.4c01407, PMID: 39158929.

(S5) Christofi, E.; Bačová, P.; Harmandaris, V. A. Physics-Informed Deep Learning Approach for Reintroducing Atomic Detail in Coarse-Grained Configurations of Multiple Poly(lactic acid) Stereoisomers. *Journal of Chemical Information and Modeling* **2024**, *64*, 1853–1867, DOI: 10.1021/acs.jcim.3c01870.

(S6) Rubinstein, M.; Colby, R. H. *Polymer physics*; Oxford University Press, 2003.

(S7) Guseva, D.; Lazutin, A.; Vasilevskaya, V. Atomistic simulation of poly (lactic acid) of different regioregularity. *Polymer* **2021**, *221*, 123577, DOI: 10.1016/j.polymer.2021.123577.

(S8) Hsu, H.-P.; Paul, W.; Binder, K. Standard Definitions of Persistence Length Do Not

Describe the Local "Intrinsic" Stiffness of Real Polymer Chains. *Macromolecules* **2010**, *43*, 3094–3102, DOI: 10.1021/ma902715e.

(S9) Wittmer, J. P.; Beckrich, P.; Meyer, H.; Cavallo, A.; Johner, A.; Baschnagel, J. Intramolecular long-range correlations in polymer melts: The segmental size distribution and its moments. *Phys. Rev. E* **2007**, *76*, 011803, DOI: 10.1103/PhysRevE.76.011803.

(S10) Wittmer, J. P.; Meyer, H.; Baschnagel, J.; Johner, A.; Obukhov, S.; Mattioni, L.; Müller, M.; Semenov, A. N. Long Range Bond-Bond Correlations in Dense Polymer Solutions. *Phys. Rev. Lett.* **2004**, *93*, 147801, DOI: 10.1103/PhysRevLett.93.147801.

(S11) Salerno, K. M.; Bernstein, N. Persistence Length, End-to-End Distance, and Structure of Coarse-Grained Polymers. *Journal of Chemical Theory and Computation* **2018**, *14*, 2219–2229, DOI: 10.1021/acs.jctc.7b01229.

(S12) Unidad, H. J.; Goad, M. A.; Bras, A. R.; Zamponi, M.; Faust, R.; Allgaier, J.; Pyckhout-Hintzen, W.; Wischnewski, A.; Richter, D.; Fetters, L. J. Consequences of Increasing Packing Length on the Dynamics of Polymer Melts. *Macromolecules* **2015**, *48*, 6638–6645, DOI: 10.1021/acs.macromol.5b00341.

(S13) Everaers, R.; Karimi-Varzaneh, H. A.; Fleck, F.; Hojdis, N.; Svaneborg, C. Kremer-Grest Models for Commodity Polymer Melts: Linking Theory, Experiment, and Simulation at the Kuhn Scale. *Macromolecules* **2020**, *53*, 1901–1916, DOI: 10.1021/acs.macromol.9b02428.

(S14) Othman, N. Rheology and processing of poly(lactides) and their enantiomeric copolymers and blends. Ph.D. thesis, University of British Columbia, 2012.

(S15) Rawat, N.; Biswas, P. Size, shape, and flexibility of proteins and DNA. *The Journal of Chemical Physics* **2009**, *131*, 165104, DOI: 10.1063/1.3251769.

(S16) Paciolla, M.; Likos, C. N.; Moreno, A. J. Validity of Effective Potentials in Crowded Solutions of Linear and Ring Polymers with Reversible Bonds. *Macromolecules* **2022**, *55*, 2659–2674, DOI: 10.1021/acs.macromol.1c02610.

(S17) Rawat, N.; Biswas, P. Shape, flexibility and packing of proteins and nucleic acids in complexes. *Phys. Chem. Chem. Phys.* **2011**, *13*, 9632–9643, DOI: 10.1039/C1CP00027F.

(S18) Formanek, M.; Rovigatti, L.; Zaccarelli, E.; Sciortino, F.; Moreno, A. J. Gel Formation in Reversibly Cross-Linking Polymers. *Macromolecules* **2021**, *54*, 6613–6627, DOI: 10.1021/acs.macromol.0c02670.

(S19) Kalyuzhnyi, O.; Ilnytskyi, J. M.; Holovatch, Y.; von Ferber, C. Universal shape characteristics for the mesoscopic star-shaped polymer via dissipative particle dynamics simulations. *Journal of Physics: Condensed Matter* **2018**, *30*, 215101, DOI: 10.1088/1361-648X/aabc16.

(S20) Gkolfi, E.; Bačová, P.; Harmandaris, V. Size and Shape Characteristics of Polystyrene and Poly(ethylene oxide) Star Polymer Melts Studied By Atomistic Simulations. *Macromolecular Theory and Simulations* **2021**, *30*, 2170001, DOI: https://doi.org/10.1002/mats.202170001.

(S21) Behbahani, A. F.; Schneider, L.; Rissanou, A.; Chazirakis, A.; Bačová, P.; Jana, P. K.; Li, W.; Doxastakis, M.; Polińska, P.; Burkhart, C.; Müller, M.; Harmandaris, V. Dynamics and rheology of polymer melts via hierarchical atomistic, coarse-grained, and slip-spring simulations. *Macromolecules* **2021**, *54*, 2740–2762, DOI: 10.1021/acs.macromol.0c02583.

(S22) Bačová, P.; Li, W.; Behbahani, A. F.; Burkhart, C.; Polińska, P.; Doxastakis, M.; Harmandaris, V. Coupling between Polymer Conformations and Dynamics Near Amor-

phous Silica Surfaces: A Direct Insight from Atomistic Simulations. *Nanomaterials* **2021**, *11*, 2075, DOI: 10.3390/nano11082075.

(S23) Newberry, R. W.; Raines, R. T. n$\rightarrow \pi^*$ interactions in poly(lactic acid) suggest a role in protein folding. *Chem. Commun.* **2013**, *49*, 7699–7701, DOI: 10.1039/C3CC44317E.

(S24) Bartlett, G.; Choudhary, A.; Raines, R.; Woolfson, D. n$\rightarrow \pi^*$ Interactions in Proteins. *Nature chemical biology* **2010**, *6*, 615–20, DOI: 10.1038/nchembio.406.
